# *IRF5 *expression profiling in SLE patients: new leads to a pathogenic signature?

**DOI:** 10.1186/ar3966

**Published:** 2012-09-27

**Authors:** BJ Barnes, RC Stone, P Du, D Feng

**Affiliations:** 1 New Jersey Medical School, UMDNJ, Newark, NJ, USA; 2 New Jersey Medical School - University Hospital Cancer Center, UMDNJ, Newark, NJ, USA; 3 High Performance and Research Computing, UMDNJ, Newark, NJ, USA

## 

Joint linkage and association analysis from multiple laboratories have identified and confirmed polymorphisms in the interferon regulatory factor 5 (*IRF5*) gene that are statistically associated with susceptibility to systemic lupus erythematosus (SLE). *IRF5 *SNPs and genetic variants have been consistently replicated and shown to confer risk for or protection from the development of SLE. IRF5 expression is significantly upregulated in SLE patients and upregulation associates with *IRF5*-SLE risk haplotypes. *IRF5 *alternative splicing has also been shown to be elevated in SLE patients. Given that human *IRF5 *exists as multiple alternatively spliced transcripts, each with potentially distinct function(s), it is important to determine whether the *IRF5 *transcript profile expressed in healthy donor immune cells is different from that expressed in SLE patients. Moreover, the exact functional consequence of an *IRF5*-SLE risk haplotype on the profile of *IRF5 *transcripts expressed needs to be addressed. Using a combination of standard molecular cloning techniques and recent advances in next-generation sequencing technologies, we examined the profile of *IRF5 *transcripts expressed in purified immune cells of healthy donors and SLE patients. By enriching for *IRF5*, we obtained an unprecedented coverage depth >3,000-fold per sample. Molecular cloning was used to generate a full-length *IRF5 *variant transcriptome for the analysis of *IRF5 *transcript expression in primary immune cells of SLE patients and healthy donors. This method of analysis was compared with other methods that do not rely on the knowledge of an *IRF5 *transcriptome; that is, using junction counts and *de novo *junction discovery. Data from these studies support the overall complexity of *IRF5 *alternative splicing in SLE and resulted in the identification and isolation of 14 new differentially spliced *IRF5 *transcripts. Results from next-generation sequencing correlated with cloning and gave similar abundance rankings in SLE patients, thus supporting the use of this new technology for in-depth single gene transcript profiling. This study provides the first proof that SLE patients express an *IRF5 *transcript signature that is distinct from healthy donors, and that an *IRF5*-SLE risk haplotype defines the most abundant *IRF5 *transcripts expressed in SLE patients. We posit this newly defined workflow of next-generation sequencing for the rapid enrichment, identification, and quantification of differentially spliced transcripts in donor RNA samples.

